# Topology and temperature dependence of the diffuse X-ray scattering in Na_0.5_Bi_0.5_TiO_3_ ferroelectric single crystals

**DOI:** 10.1107/S160057671501571X

**Published:** 2015-09-20

**Authors:** Semën Gorfman, Dean S. Keeble, Alessandro Bombardi, Pam A. Thomas

**Affiliations:** a Department of Physics, University of Siegen, Walter-Flex Strasse 3, Siegen 57072, Germany; b Diamond Light Source, Harwell Science and Innovation Campus, Didcot OX11 0DE, UK; c Department of Physics, University of Warwick, Gibbet Hill Road, Coventry CV4 7AL, UK

**Keywords:** single-crystal diffuse X-ray scattering, lead-free ferroelectrics, high-resolution X-ray diffraction, perovskites, Na_0.5_Bi_0.5_TiO_3_

## Abstract

The results of high-resolution measurements of the diffuse X-ray scattering produced by a perovskite-based Na_0.5_Bi_0.5_TiO_3_ ferroelectric single crystal between 40 and 620 K are reported. The study was designed as an attempt to resolve numerous controversies regarding the average structure of Na_0.5_Bi_0.5_TiO_3_, such as the mechanism of the phase transitions between the tetragonal, *P*4*bm*, and rhombohedral | monoclinic, *R*3*c* | *Cc*, space groups and the correlation between structural changes and macroscopic physical properties. The starting point was to search for any transformations of structural disorder in the temperature range of thermal depoling (420–480 K), where the average structure is known to remain unchanged. The intensity distribution around the {032} pseudocubic reflection was collected using a PILATUS 100K detector at the I16 beamline of the Diamond Light Source (UK). The data revealed previously unknown features of the diffuse scattering, including a system of dual asymmetric L-shaped diffuse scattering streaks. The topology, temperature dependence, and relationship between Bragg and diffuse intensities suggest the presence of complex microstructure in the low-temperature *R*3*c* | *Cc* phase. This microstructure may be formed by the persistence of the higher-temperature *P*4*bm* phase, built into a lower-temperature *R*3*c* | *Cc* matrix, accompanied by the related long-range strain fields. Finally, it is shown that a correlation between the temperature dependence of the X-ray scattering features and the temperature regime of thermal depoling is present.

## Abstract 2

The high-resolution temperature-dependent diffuse X-ray scattering from an Na_0.5_Bi_0.5_TiO_3_ perovskite-based single crystal is measured on the approach to the polymorphic phase transition. The previously unseen topological features of the diffuse scattering and their temperature evolution are reported.

## 1. Introduction   

Na_0.5_Bi_0.5_TiO_3_ (NBT) is a ferroelectric material that is interesting both as the foundation of many potential lead-free piezoelectrics (Takenaka *et al.*, 2008 ▸) and as a model system for the crystallography of perovskites (Mitchell, 2003 ▸). NBT is unusual in showing at least two polymorphic structural phase transformations (Vakhrushev *et al.*, 1985 ▸; Park *et al.*, 1996 ▸; Jones & Thomas, 2002 ▸), formation of hierarchical domain systems and peculiar structural disorder (Yao *et al.*, 2010 ▸; Levin & Reaney, 2012 ▸). Despite a significant number of X-ray and neutron diffraction studies (Jones & Thomas, 2000 ▸; Jones & Thomas, 2002 ▸; Aksel, Forrester, Kowalski *et al.*, 2011 ▸; Ge *et al.*, 2013 ▸; Rao, Fitch & Ranjan, 2013 ▸; Rao, Datta *et al.*, 2013 ▸; Carter *et al.*, 2014 ▸), electron microscopy investigations (Beanland & Thomas, 2011 ▸; Dorcet & Trolliard, 2008 ▸; Dorcet *et al.*, 2008 ▸; Liu *et al.*, 2012 ▸; Ma *et al.*, 2013 ▸) and density functional theory calculations (Gröting *et al.*, 2011 ▸, 2012 ▸; Meyer *et al.*, 2015 ▸), considerable disagreement about the average structure of NBT remains.

The commonly accepted reference point stems from the seminal neutron powder diffraction study of Jones & Thomas (2002 ▸). They reported two temperature-induced phase transitions, involving changes of average symmetry, realignment of polarization and the modification of oxygen octahedral tilt systems: (*a*) *T* ≃ 840 K, the transition between the cubic paraelectric, 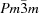, phase and tetragonal ferro(i)electric, *P*4*bm*, phase with the 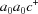 octahedral tilt system according to the Glazer (1972 ▸) notation; (*b*) *T* ≃ 570 K, the transition between tetragonal ferro(i)electric, *P*4*bm*, and rhombohedral ferroelectric, *R*3*c*, phases (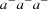 octahedral tilt system). Despite the wide acceptance of this phase pattern as a framework, the fine structural details, especially those in the nominally rhombohedral *R*3*c* phase, are still the matter of revisions and apparent contradictions. Even the original work of Jones & Thomas (2002 ▸) documented local departures from the *R*3*c* average structure in the form of significant disorder of the Bi positions. Furthermore, the mechanism of transition between the structures described by the *R*3*c* and *P*4*bm* space groups, which lack a group–subgroup relation, remains unexplained.

A rapid reassessment of the NBT average structure began from the single-crystal X-ray diffraction work of Gorfman & Thomas (2010 ▸) and synchrotron X-ray powder diffraction work of Aksel, Forrester, Jones *et al.* (2011 ▸). Benefiting from the capabilities of modern instruments, both studies independently presented evidence of a lower-symmetry long-range monoclinic phase, with 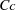 rather than *R*3*c* symmetry. Subsequently, Ma *et al.* (2013 ▸) have confirmed the long-range *Cc* symmetry of NBT by careful analysis of the half-integer pseudocubic reflections, using transmission electron microscopy. They noted, however, that the NBT structure evolves into *R*3*c* after a small addition of BaTiO_3_. Rao & Ranjan (2012 ▸) and Rao, Fitch & Ranjan (2013 ▸) suggested a coexistence of monoclinic and rhombohedral phases below ∼570 K and reported that the monoclinic phase, *Cc*, can be irreversibly driven into the rhombohedral *R*3*c* phase by the application of an external electric field. Observation of optical birefringence in NBT single crystals by Gorfman *et al.* (2012 ▸) supplied further evidence of the monoclinic symmetry of the lower-temperature NBT phase. This work reported a ∼2 (1) K temperature window of optically isotropic phase just below the phase transition at 573 (20) K, which remains unexplained and suggests hidden and complex mechanisms driving this phase transition. However, Beanland & Thomas (2014 ▸) have carefully inspected NBT using a novel computer-controlled electron microscopy technique and concluded that any defect-free areas of NBT must be described by the *R*3*c* symmetry. The apparent bulk monoclinic symmetry of NBT was questioned by Ge *et al.* (2013 ▸), who suggested that it must be rather attributed to a skin effect. Considering all the existing controversies we will refer to the low-temperature phase here as 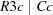.

Another puzzling problem is the mismatch between the reported temperature dependence of the average structure and physical properties: the piezoelectricity in poled NBT ceramics is irreversibly lost upon heating at ∼420–480 K (Hiruma *et al.*, 2009 ▸; Aksel *et al.*, 2012 ▸; Foronda *et al.*, 2014 ▸). None of the studies report any abrupt structural changes at these temperatures. Only recently, Rao, Datta *et al.* (2013 ▸) performed temperature-dependent neutron powder diffraction on poled NBT ceramics and suggested that the onset of thermal depoling might be caused by the appearance of regions with octahedral tilt systems matching that of the tetragonal symmetry, *P*4*bm*. Thus, the properties of NBT might be influenced by the local structural disorder and microstructure: the parameters which go beyond the average atomic structure.

**Figure 1:**
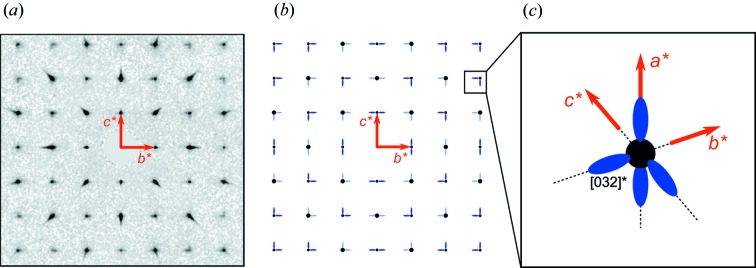
An overview of the previously known information about the topology of the L-shaped DS streaks in NBT. (*a*) 0*kl* section of reciprocal space reconstructed from the data collected using a home-laboratory Gemini R diffractometer. (*b*) A schematic diagram showing the topology of the diffuse scattering streaks (blue) in relation to the Bragg scattering (black). (*c*) An enlarged three-dimensional schematic view of a smaller reciprocal space volume. The streaks extend along one of the 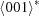 reciprocal lattice directions.

Indeed, NBT is known for a significant structural disorder. For example, the shortest Bi—O bond distance, calculated from the average structure as 2.53 Å, disagrees with the 2.2 Å bonds observed with EXAFS (Shuvaeva *et al.*, 2005 ▸). Keeble *et al.* (2013 ▸) and Aksel *et al.* (2013 ▸) analysed the total neutron scattering by means of reverse Monte Carlo simulation, concluding that accounting for total neutron scattering instead of isolated Bragg peaks resolves this controversy. Further direct evidence of structural disorder is provided by single-crystal diffuse X-ray scattering (DS) (Kreisel *et al.*, 2003 ▸; Thomas *et al.*, 2010 ▸) and singe-crystal neutron scattering data (Balagurov *et al.*, 2006 ▸; Ge *et al.*, 2013 ▸). Although strong DS is quite common for perovskite-based ferroelectrics and relaxor ferroelectric single crystals (*e.g.* Xu *et al.*, 2006 ▸; Stock *et al.*, 2007 ▸; Paściak *et al.*, 2012 ▸; Bosak *et al.*, 2012 ▸), X-ray DS in NBT is highly unusual. It exhibits a scheme of asymmetric DS streaks, emanating from the Bragg peaks and generally extending along the lower-angular 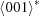 directions (the pseudocubic cell setting is used for indexing throughout this study). More specifically, 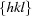 reflections (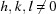) are decorated by three DS streaks, extending towards decreasing absolute values of *|h|*, *|k|* and *|l|*; 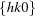 reflections (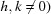 are decorated by four streaks: one each extending towards decreasing *|h|* and *|k|*, and two others extending towards increasing *|l|*; 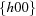 reflections (*h ≠ 0*) are decorated by four DS streaks towards increasing *|k|* and *|l|*. The intensity of these streaks is *hkl* dependent: for example, they are particularly strong around {032}-type peaks and can be easily observed using a laboratory-based single-crystal X-ray diffractometer, whereas they are barely visible around *e.g.* {110}-type peaks. Fig. 1 ▸ shows (*a*) typical diffraction data as observed using a laboratory diffractometer, along with (*b*) a schematic of the DS in the 0*kl* plane and (*c*) a schematic of the DS distribution around the [032]* reciprocal lattice point, highlighting the asymmetry of the DS. Kreisel *et al.* (2003 ▸) modelled this DS by including planar ‘island’ sheets in the rhombohedral ‘matrix’ of NBT, where Bi and Na atoms are displaced in 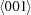 directions off the threefold 〈111〉 axis. Although this model predicts the appearance of asymmetric DS streaks, it fails to reproduce the exact topology observed, *e.g.* the presence of local intensity maxima along the streaks (Thomas *et al.*, 2010 ▸). It is also only an empirical model and therefore contains no intrinsic chemical or physical reasoning for the formation of such planar sheets. Furthermore, it is not known how the DS changes with temperature and, in contrast to the average structure, whether or not there is evidence in the DS of a structural transition that accompanies the loss of piezoelectric properties at 420–480 K. Finally, Ge *et al.* (2013 ▸) reported that neutron diffuse scattering is qualitatively different from X-ray diffuse scattering: while weak 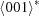-oriented streaks are observed using neutron scattering, their distribution around Bragg peaks exhibits twofold rotational symmetry.

The first aim of this work is to reinvestigate the exact topology of the X-ray DS by using a state-of-the-art high-resolution synchrotron X-ray diffractometer. The second aim is to probe the temperature dependence of this DS to assess the role of the structural disorder in the physical properties of NBT. Finally, we examine the role of structural disorder in the mechanism of the phase transition between the 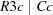 and *P*4*bm* phases. Understanding this mechanism in NBT may advance the understanding of structural mechanisms of phase transition in other functional ferroelectrics such as lead zirconate titanate (PbZr_1−*x*_Ti_*x*_O_3_), as well as popular solid solutions of NBT such as Na_0.5_Bi_0.5_TiO_3_–BaTiO_3_ (NBT–BT).

## 2. Experiment   

High-resolution measurements of the X-ray diffuse scattering were performed at the I16 beamline at Diamond Light Source, UK. An NBT single crystal was grown by the flux method as previously described by Jones & Thomas (2000 ▸). A fragment of straw-coloured crystal was selected, oriented and cut parallel to one of the pseudo-cubically equivalent (011) Miller planes. The crystal of approximately 1 × 1 × 0.1 mm was attached to a silicon wafer using conductive silver paint and then mounted in a Lakeshore cryofurnace. The orientation of the crystal on the silicon wafer ensured easy access to one of the {032} reflections on the I16 six-circle diffractometer. We collected reciprocal space volumes around the 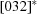 point of reciprocal space and in the temperature range 40–620 K (*i.e.* up to and beyond the transition to the tetragonal phase at 573 (20) K (Gorfman *et al.*, 2012 ▸) on both heating and cooling. The maps were taken every 20 K below 400 K, every 5 K between 400 and 560 K, and every 1 K above 560 K. We tuned the X-ray wavelength/energy to 0.93 Å/13.3 keV: this is close to the Bi *L*_III_ absorption edge (0.92 Å/13.4 keV) to balance reciprocal space resolution and attenuation. At this energy and for the geometry of the 032 reflection, the average X-ray penetration depth was 19.7 µm. The scattered intensity in each slice of reciprocal space was measured using a PILATUS 100K detector (DECTRIS), and a reciprocal space volume was built up by taking sequential frames every 0.02° in ω. The resolution in 2θ and χ was set by the pixel size of the PILATUS to ∼0.015°. This experimental methodology resulted in sampling reciprocal space with a resolution of ∼0.0015 pseudocubic reciprocal lattice units (r.l.u.). Furthermore, we benefited from the high dynamic range of the PILATUS (Kraft *et al.*, 2009 ▸) to capture the intensity extending over strong Bragg and weak diffuse scattering signal.

## 3. Results   

### 3.1. Topology of the L-shaped diffuse scattering   

**Figure 2:**
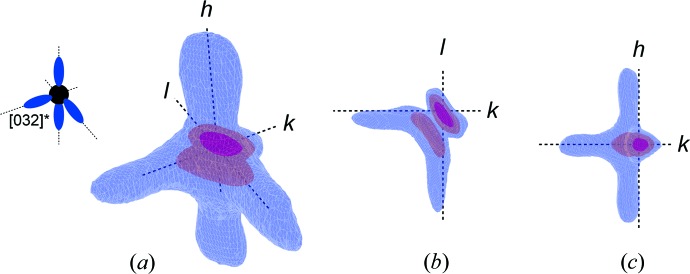
Three-dimensional reconstructions of the DS around the 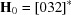 point of the reciprocal lattice. (*a)* represents the schematic view of the DS scattering around this point of the reciprocal lattice and reconstructs the three-dimensional contour of the present high-resolution I16 DS measurement. (*b*), (*c*) represent the same contours, using different projections. An animated three-dimensional version of this reciprocal space volume is available in the supporting information.

Fig. 2 ▸ shows the reconstructed three-dimensional reciprocal space volume (RSV), with isosurfaces of three-dimensional scattering intensity distribution, 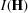, around the 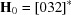 reciprocal lattice point, collected at a temperature of 40 K. Fig. 2 ▸(*a*) compares the schematics of the RSV from the previous lower-resolution experiments with the result of the presented high-resolution experiments. Figs. 2 ▸(*b*) and 2 ▸(*c*) view the RSV along the [100]* and [001]* directions, respectively. The detailed RSV (in animated form) is available in the supporting information.

**Figure 3:**
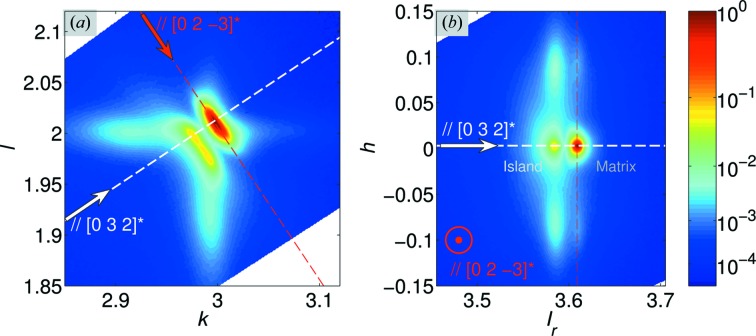
The crystallographic orientation of the observed DS. (*a*) A section parallel to 0*kl* in the crystallographic reference frame in which the data were collected. The arrows indicate the radial (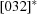) and transverse (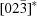) crystallographic directions. (*b*) The 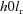 plane, where 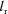 is the projection of the scattering vector onto the 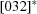 direction. The plane is perpendicular to the 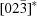 direction. Both orientations highlight the low-intensity plane appearing perpendicular to the 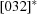 direction. Such low-intensity planes are a typical feature of Huang scattering: diffuse X-ray scattering due to the presence of long-range deformation fields, centred by point defects.

The collection of high-resolution RSVs gives us the added ability to extract reciprocal space maps (RSMs) from arbitrary planes, two of which are shown in Fig. 3 ▸. These RSMs highlight the fine details of the DS topology: they pass through the maximum of the three-dimensional scattering intensity and are parallel to the 0*kl* (Fig. 3 ▸ *a*) and 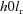 (Fig. 3 ▸ *b*) reciprocal space planes, where 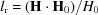, *i.e.* the projection along the line connecting the origin of reciprocal space to the **H**_0_ = 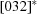 reciprocal lattice point. These RSMs reveal previously unseen features of the DS, which are described below.

#### 3.1.1. DS Streaks   

There are two ‘systems’ of 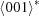 DS streaks: stronger streaks extend towards the lower scattering angles (larger *d* spacing), whilst weaker streaks extend towards the higher scattering angles (smaller *d* spacing). The stronger streaks were observed in all the previous measurements (Fig. 1 ▸). However, this is the first observation of the weaker streaks, which have never been accounted for in any model describing structural disorder in perovskite-based materials.

#### 3.1.2. Bragg peaks   

The Bragg peak is split into two components: a stronger component (later referred to as the ‘matrix’ peak) and a second peak of weaker intensity closer to the origin (later referred to as the ‘island’ peak), separated from the stronger component by a plane of low intensity. Careful inspection of the temperature dependence of these peaks will suggest that their splitting cannot be accounted for by twinning/ferroelastic domains.

#### 3.1.3. Relationship between the diffuse scattering streaks and the Bragg peaks   

We see that the stronger DS streaks decorate the ‘island’ peak, whilst the weaker DS streaks decorate the ‘matrix’ peak. The two ‘Bragg’ peaks are separated by a plane of low-intensity scattering, which appears perpendicular to the 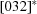 reciprocal lattice direction. This feature is reminiscent of the plane of low intensity that results from so-called Huang scattering (Ekstein, 1945 ▸; Krivoglaz, 1996 ▸). Huang scattering is the DS produced by long-range elastic fields created by point defects in a crystal. For example, Huang scattering has been recently reported in the DS from single crystals of PbZr_1−*x*_Ti_*x*_O_3_ (Burkovsky *et al.*, 2012 ▸). The presence of the low-intensity plane suggests that the observed DS from NBT might occur as a result of a similar type of long-range elastic strain field. We should note, however, that there is an important difference between the observed DS and Huang scattering: Huang scattering is symmetrically distributed around a Bragg node, whereas the observed DS in NBT is not.

### 3.2. Temperature dependence of the scattering intensity   

**Figure 4:**
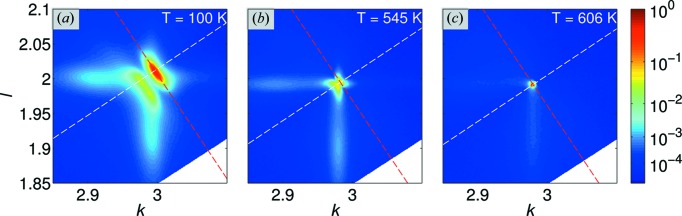
The temperature dependence of the RSMs around the 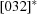 reciprocal lattice point, as seen on the 0*kl* plane in Fig. 3 ▸(*a*). The first two temperatures, (*a*) 100 K and (*b*) 545 K, correspond to the lower-temperature 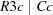 phase; (*c*) 606 K corresponds to the tetragonal *P*4*bm* phase. The white and red dashed lines show the crystallographic 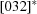 and 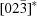 directions, respectively [as in Fig. 3 ▸(*a*)]. An animated version of this figure is available in the supporting information.

The observed intensity distributions are temperature dependent. Fig. 4 ▸ displays the RSMs [the same section as in Fig. 3 ▸(*a*)] at three temperatures upon heating: 100 K [below the 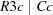
–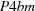 phase transition; Fig. 4 ▸(*a*)], 545 K [on approach to the phase transition; Fig. 4 ▸(*b*)] and 606 K [above the phase transition; Fig. 4 ▸(*c*)]. An animation of the temperature dependence of the RSVs (on both heating and cooling) is available in the supporting information. It shows that all of the scattering described above becomes weaker at higher temperature and below the phase transition. The RSV collected above the phase transition consists of a single Bragg reflection decorated by some very weak DS streaks that extend towards the lower scattering angles; this Bragg peak appears extremely sharp, with a full width at half-maximum of only 0.003 r.l.u. In order to describe quantitatively the thermal evolution of the scattering we will discuss now in turn the behaviours of the Bragg and diffuse scattering features.

#### 3.2.1. Temperature dependence of the Bragg scattering: the evidence for low-temperature phase segregation   

**Figure 5:**
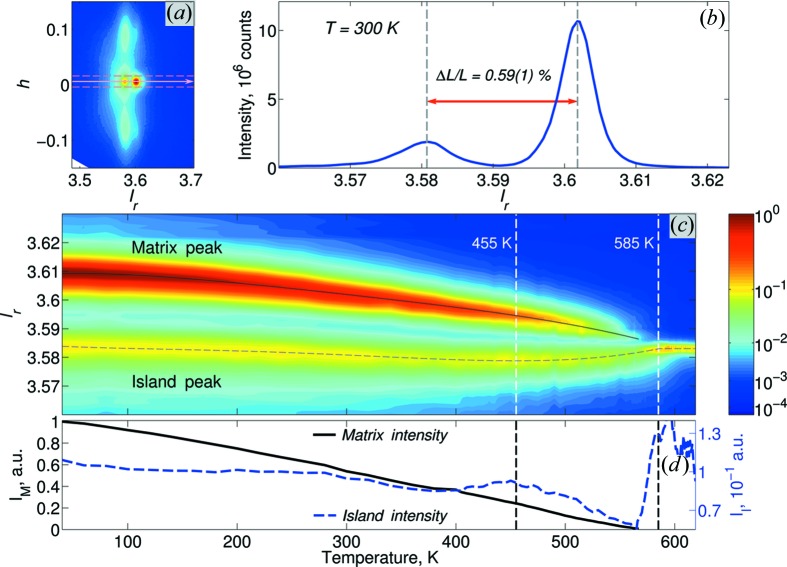
The temperature dependence of the radial scattering intensity profile along the 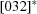 direction. (*a*) The *h*0*l*_r_ reciprocal space map, showing the radial direction along which the profile is taken (arrow) and the corresponding integration box (dashed lines); (*b*) the resulting intensity profile for the 300 K data; (*c*) a false-colour map showing the temperature dependence of the radial profile, the extracted peak positions being highlighted by the solid and dashed lines; (*d*) the extracted peak intensities. The nominal temperature of the phase transition is shown by the vertical dashed line at 585 K; the average temperature of thermal depoling (known from previous work) is shown by the vertical dashed line at 455 K. The intensity of the matrix peak can only be followed until 570 K.

Fig. 5 ▸ summarizes the temperature dependence of the radial intensity profile, reconstructed along the 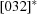 reciprocal lattice direction and passing through the overall intensity maximum. For each temperature the one-dimensional radial intensity, 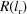, is reconstructed from the three-dimensional data as 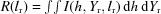, where 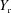 represents the coordinate parallel to the 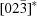 reciprocal lattice direction. The integration was carried out over a 0.02 × 0.02 r.l.u. square. The radial direction 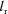 is shown by the arrow in Fig. 5 ▸(*a*), the borders of the integration are shown by the dashed lines, and the resultant radial profile for the 300 K data is shown in Fig. 5 ▸(*b*). The thermal evolution of this profile describes the radial splitting of the Bragg reflection as a function of temperature (Fig. 5 ▸ *c*).

Fig. 5 ▸(*c*) shows that far from the phase transition both island and matrix Bragg peaks move to larger *d* spacing (lower 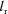 values) upon heating, which is consistent with thermal expansion. However, at ∼455 K (*i.e.* ∼120 K below the phase transition), some peculiarities appear: firstly, the position of the island peak reaches a point of inflection and begins to behave as though the corresponding sample volumes had negative thermal expansion. This movement allows the island and matrix Bragg peaks to approach each other, yet as they do so the matrix peak disappears. The island peak continues into the high-temperature phase and becomes the very sharp single Bragg reflection associated with the tetragonal structure. Fig. 5 ▸(*d*) shows the details of the temperature dependence of the matrix and island intensities (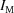 and 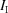), reconstructed along the dashed lines in Fig. 5 ▸(*c*). It shows that the matrix peak decays linearly with the temperature, becoming immeasurably small a few kelvin before the nominal phase transition temperature. At the same time the island peak reduces by just 50% until it flows into the single sharp Bragg peak observed in the tetragonal phase.

Although the temperature dependence of the matrix and island peak positions looks similar to the behaviour expected for a ferroelastic phase transition, the temperature dependence of their intensities and the presence of diffuse scattering indicate a more complex picture. The continuation of the high-temperature Bragg peak into the island Bragg peak at lower temperatures and the absence of the matrix peak a few kelvin before the phase transition suggest that the island peak is a persistence of the tetragonal phase below the transition. We must assume some phase segregation, so that matrix peaks are diffracted by the dominating volumes of monoclinic/rhombohedral phase, whereas island peaks are diffracted from the tetragonal inclusions, remnants of the high-temperature tetragonal phase. Considering the lattice mismatch between higher- and lower-temperature phases we must also assume that such phase separation creates long-range deformation fields and produces the characteristic diffuse scattering pattern.

Note that similar phase segregation/local microstructure models in NBT appear in the literature. For example, it resembles the model of tetragonal inclusions in a rhombohedral matrix suggested as a result of transmission electron microscopy imaging of NBT (Beanland & Thomas, 2011 ▸; Yao *et al.*, 2012 ▸). It agrees with the observation of Rao, Datta *et al.* (2013 ▸) and Liu *et al.* (2012 ▸), who reported the appearance of in-phase octahedral tilt regions (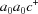 octahedral tilt systems), consistent with tetragonal *P*4*bm* symmetry, in the 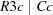 phase. Similar formation of 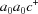 planar defects was reported in 0.96NBT–0.04BT single crystals (Daniels *et al.*, 2011 ▸, 2012 ▸), where 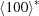 diffuse scattering rods were observed around half-integer reflections. Such planar defects were described as ‘stacking faults’, separating two rhombohedral 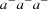 domains. The tetragonal symmetry in such ‘stacking faults’ was also demonstrated by Beanland (2011 ▸). Alternatively, the segregations in the lower-temperature phase of NBT have been also discussed by Gröting *et al.* (2011 ▸), who performed first-principle calculations to test the possible chemical ordering on the *A* site and suggested that such short-range ordering (with alternating layers of Bi and Na along 〈100〉) is possible below ∼570 K and could provide nucleation sites for larger regions.

#### 3.2.2. Thermal expansion coefficients of the matrix and island phases   

**Figure 6:**
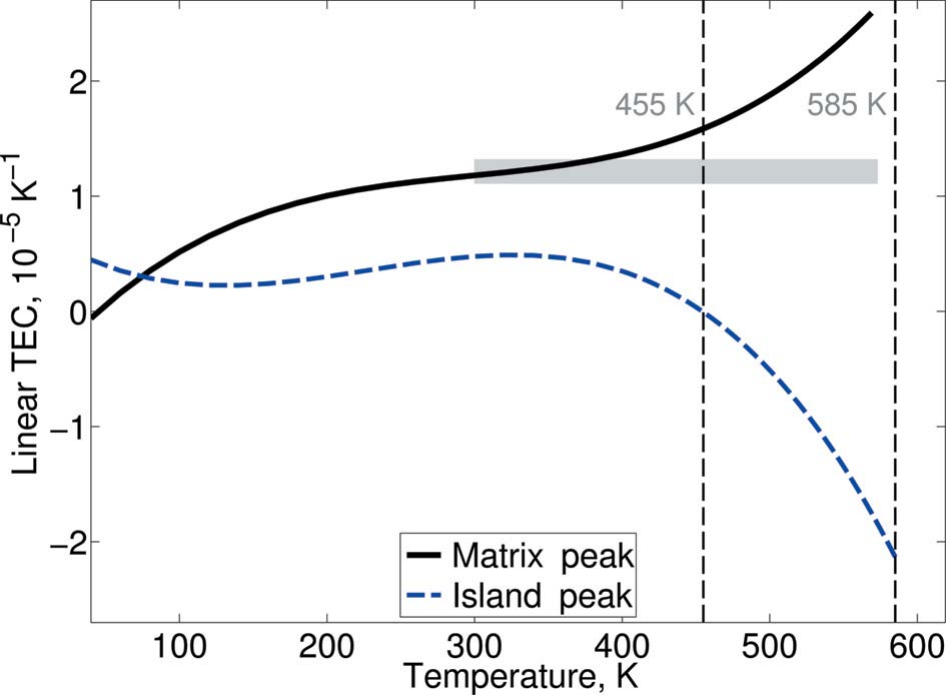
The linear thermal expansion coefficient (TEC), calculated using the positions of the matrix and island peaks. The range of TEC coefficients reported by Park *et al.* (1996 ▸) is marked by the grey rectangle.

This assumption of phase segregation below the phase transition is further supported by some simple macroscopic strain analysis. The relative separation between the island and matrix Bragg peaks at 300 K (Fig. 5 ▸ *b*) is 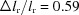
 (4)%. It matches the separation between the lattice parameters of the room-temperature phase and the lattice parameters of the high-temperature tetragonal phase reported by Jones & Thomas (2002 ▸): 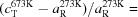 0.52 (1)% [here 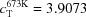
 (2) Å is the tetragonal *c* lattice parameter at 673 K and 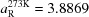
 (2) Å is the pseudocubic *a* lattice parameter at room temperature]. We elaborated on this phase segregation idea by analysing the linear thermal expansion corresponding to each of the above phases. This thermal expansion was calculated according to 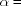 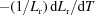 [*L*_r_ denotes the positions of the maxima of the peak(s)] and is shown in Fig. 6 ▸. The individual thermal behaviours of the phases differ. The position of the island peak appears almost temperature independent; it exhibits little overall thermal expansion between 40 and 573 K, reaching the maximum of 0.45 × 10^−5^ K^−1^ at around 320 K. The position of the matrix peak adopts a more typical thermal expansion over this temperature range; it is comparable to the thermal expansion previously reported for NBT single crystals (1.50–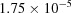
 K^−1^) according to Park *et al.* (1996 ▸). This behavioural difference diverges further as the phase transition is approached, as the matrix peak enters a region of negative thermal expansion as previously mentioned. The systematically lower thermal expansion of the tetragonal island phase suggests that the regions of this symmetry might be trapped by the monoclinic/rhombohedral structure that develops.

#### 3.2.3. Temperature dependence of the diffuse streaks   

**Figure 7:**
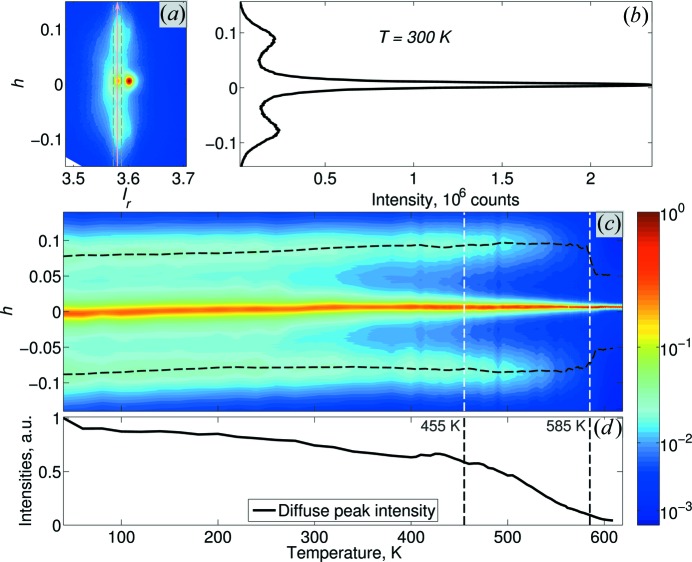
The temperature dependence of the diffuse scattering intensity profile, 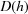, along the [100]* direction. (*a*) The *h*0*l*_r_ reciprocal space map, showing the direction along which the 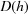 profile is taken (arrow) and the corresponding integration box (dashed lines); (*b*) the resulting intensity profile for the 300 K data; (*c*) false-colour map showing the temperature dependence of the intensity profile, the extracted peak positions of the DS streaks being highlighted by the dashed lines; (*d*) the extracted peak intensities (averaged over both streaks). The vertical dashed lines in both (*c*) and (*d*) mark the same temperatures as in Fig. 5 ▸.

Fig. 7 ▸ (organized in a similar way to Fig. 5 ▸) shows the temperature dependence of the diffuse scattering. The intensity profile in Fig. 7 ▸(*b*) is reconstructed from the three-dimensional data as 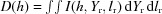, where the integration was carried out within a 0.015 × 0.015 r.l.u. box. Fig. 7 ▸(*a*) indicates the *h* direction and the corresponding integration box with an arrow and dashed lines, respectively. Fig. 7 ▸(*b*) shows the as-defined profile for the 300 K data. The temperature dependence of this profile is then displayed in Fig. 7 ▸(*c*). Fig. 7 ▸(*d*) follows the intensity of the diffuse streaks, 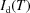, reconstructed along their maxima [shown by the dashed lines in Fig. 7 ▸(*c*)]. The intensity of the DS streaks decreases as the phase transition is approached, in a fashion very reminiscent of the matrix Bragg peak.

#### 3.2.4. Relationship between Bragg and diffuse scattering   

**Figure 8:**
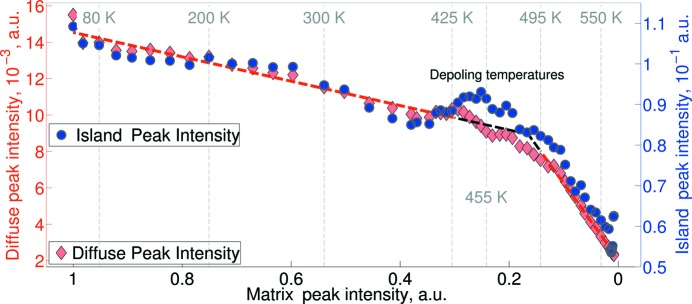
Relationships between the intensities of the matrix Bragg peak, diffuse streaks (diamonds) and island (circles) Bragg peaks. The dashed red lines display the results of the linear regression in the two different temperature ranges: below 425 K and above 495 K. The change of the linear regression coefficient connecting the intensity of the diffuse scattering as a function of the matrix peak intensity points to the ‘virtual’ transition, involving the modification of the fine disorder parameter at these temperatures.

As a last step we investigated the relationship between the temperature dependences of the island and matrix Bragg peaks and the DS. We plot all these dependencies in the temperature range below 570 K, where the intensity of both peaks is measurable. Fig. 8 ▸ shows that the functional dependence of the DS intensity on the matrix Bragg peak intensity can be described by two linear regression intervals: below ∼425 K and above ∼495 K. The change of the linear regression coefficient between these temperatures points to a possible ‘virtual’ phase transition, which describes the change of the balance between long-range-ordered structure (responsible for the Bragg peak) and structural disorder (responsible for the DS).

## 4. Conclusion   

In conclusion, we have reconstructed the fine details of single-crystal DS from NBT with a resolution of ∼10^−3^ r.l.u. Then we have carefully inspected the topology and temperature dependence of the scattering and followed their evolution on the approach to and through the 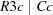 to 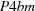 structural phase transition. Our data show that such a phase transition must generate the complex bulk microstructure of the 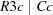 phase and a correspondingly characteristic X-ray scattering pattern: the Bragg peak in the 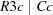 phase separates into two components, the weaker of which (‘island’ peak) behaves as a representative of the persistence of the tetragonal *P*4*bm* phase, while another, stronger peak (‘matrix’ peak) emerges just below the phase transition temperature. Both peaks are decorated by L-shaped DS streaks: the stronger streaks flow out of the island Bragg peak and extend towards lower scattering angles/higher *d* spacing; the weaker streaks flow out of the matrix Bragg peak, extending towards higher scattering angles/lower *d* spacing. The two Bragg peaks, together with their associated DS, are separated by a plane of low intensity which is reminiscent of that observed because of Huang scattering. We have also found that the relative separation between the matrix and island Bragg peaks matches the relative separation between the lattice parameters of the previously reported high-temperature (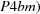 and low-temperature 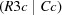 phases (Jones & Thomas, 2002 ▸).

The geometry and the temperature dependence of the scattering patterns suggest that the low-temperature phase may be a phase coexistence in which volumes of higher-temperature *P*4*bm* phase persist below the phase transition into the low-temperature 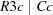 phase. Such microstructure must create a pattern of the long-range strain fields of monoclinic (*m*) point symmetry in both 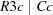 matrix and *P*4*bm* island phase components. This model may account for the multiple controversies about the structure and symmetry of the low-temperature 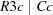 phase: ranging from purely rhombohedral *R*3*c* in the defect-free areas (Beanland & Thomas, 2014 ▸) to long-range monoclinic *Cc* (Gorfman & Thomas, 2010 ▸; Aksel, Forrester, Jones *et al.*, 2011 ▸; Ma *et al.*, 2013 ▸) and the combination of both (Rao, Datta *et al.*, 2013 ▸). Considering this model of phase coexistence, it becomes possible to explain why the apparent symmetry depends on the length scale of the probe, the type of sample, doping and even the preparation history, in which case the local concentration of tetragonal inclusions may differ from sample to sample.

We must emphasize that more specific details of the structural disorder responsible for the asymmetric L-shaped diffuse scattering remain unknown. The topological similarity of the observed DS to Huang scattering suggests that the underlying structural disorder may originate from the long-range elastic deformation field, extending both inwards and outwards from the segregated tetragonal *P*4*bm* phase discussed above. However, Huang scattering does not account for the observed asymmetry of the DS streaks. Therefore, some essential modifications of the corresponding model of the structural disorder must be considered. For example, it is known that NBT may show significant positional disorder (*e.g.* Keeble *et al.*, 2013 ▸) of the Bi atoms. We may assume that any long-range deformation field may trigger local atomic displacements, thus modifying the structural disorder. Such possible involvement of Bi displacements can be also supported by the fact that the reported X-ray diffuse scattering, which is highly dominated by Bi positions, strongly differs from neutron diffuse scattering (Ge *et al.*, 2013 ▸).

Despite remaining uncertainties, our studies evidence a ‘virtual’ phase transition that marks a change between two different temperature dependencies of the scattering intensities. The apparent match of the ‘virtual’ phase transition temperature in NBT with the temperature of thermal depoling is an interesting coincidence that merits further investigation.

## Supplementary Material

The animated three-dimensional intensity distribution around [032]* reciprocal lattice point: Camera View 1. DOI: 10.1107/S160057671501571X/ks5473sup1.wmv

The animated three-dimensional intensity distribution around [032]* reciprocal lattice point: Camera View 2. DOI: 10.1107/S160057671501571X/ks5473sup2.wmv

The animated temperature dependence of three-dimensional intensity distribution around [032]* reciprocal lattice point. DOI: 10.1107/S160057671501571X/ks5473sup3.wmv

The temperature dependence of intensity distribution: 0kl reciprocal space map view. DOI: 10.1107/S160057671501571X/ks5473sup4.wmv
